# Haloperidol-induced changes in neuronal activity in the striatum of the freely moving rat

**DOI:** 10.3389/fnsys.2013.00110

**Published:** 2013-12-16

**Authors:** Dorin Yael, Dagmar H. Zeef, Daniel Sand, Anan Moran, Donald B. Katz, Dana Cohen, Yasin Temel, Izhar Bar-Gad

**Affiliations:** ^1^The Leslie & Susan Goldschmied (Gonda) Multidisciplinary Brain Research Center, Bar-Ilan UniversityRamat-Gan, Israel; ^2^Departments of Neuroscience and Neurosurgery, Maastricht University Medical CenterMaastricht, Netherlands; ^3^Department of Psychology, Volen National Center for Complex Systems, Brandeis UniversityWaltham, MA, USA

**Keywords:** striatum, dopamine antagonist, extra cellular recording, neuronal subpopulations, oscillations

## Abstract

The striatum is the main input structure of the basal ganglia, integrating input from the cerebral cortex and the thalamus, which is modulated by midbrain dopaminergic input. Dopamine modulators, including agonists and antagonists, are widely used to relieve motor and psychiatric symptoms in a variety of pathological conditions. Haloperidol, a dopamine D2 antagonist, is commonly used in multiple psychiatric conditions and motor abnormalities. This article reports the effects of haloperidol on the activity of three major striatal subpopulations: medium spiny neurons (MSNs), fast spiking interneurons (FSIs), and tonically active neurons (TANs). We implanted multi-wire electrode arrays in the rat dorsal striatum and recorded the activity of multiple single units in freely moving animals before and after systemic haloperidol injection. Haloperidol decreased the firing rate of FSIs and MSNs while increasing their tendency to fire in an oscillatory manner in the high voltage spindle (HVS) frequency range of 7–9 Hz. Haloperidol led to an increased firing rate of TANs but did not affect their non-oscillatory firing pattern and their typical correlated firing activity. Our results suggest that dopamine plays a key role in tuning both single unit activity and the interactions within and between different subpopulations in the striatum in a differential manner. These findings highlight the heterogeneous striatal effects of tonic dopamine regulation via D2 receptors which potentially enable the treatment of diverse pathological states associated with basal ganglia dysfunction.

## Introduction

The striatum is the main input structure of the basal ganglia, a group of sub-cortical nuclei involved in motor, limbic and associative behavior. The vast majority (>95%) of striatal neurons are the GABAergic projection neurons, which are traditionally termed medium spiny neurons (MSNs) (Rymar et al., 2004). MSNs can be divided into D1-receptor expressing MSNs projecting to the substantia nigra pars reticulata (SNr), and the entopeduncular nucleus (EP) and D2-receptor expressing MSNs projecting to the globus pallidus (GP) (Albin et al., 1989). MSNs are characterized by bursty firing in response to different movements and behaviors with a very low baseline firing rate (Wilson and Groves, 1981). Additionally, the striatum contains multiple small populations of interneurons (Tepper et al., 2004). Two of the most extensively studied interneuron populations are the GABAergic fast spiking interneurons (FSIs) and the cholinergic tonically active interneurons (TANs). FSIs, which comprise ~1% of the striatal cell population, receive inputs from the cortex and GP, project to surrounding MSNs, and exert a strong inhibitory modulation (Berke, 2011). Electrophysiologically, FSIs display narrow spike waveforms with a high baseline firing rate and resemble the FSIs found in the hippocampus and cerebral cortex (Somogyi and Klausberger, 2005; Berke, 2011). TANs, which make up 1–2% of the striatal population (Goldberg and Reynolds, 2011), have a tonic irregular firing pattern, lack burst activity, and are strongly influenced by both positive and aversive stimuli. TANs show wide and high amplitude extracellular spike waveforms, with a relatively long refractory period (Sharott et al., 2009).

Dopamine and dopamine receptors play a key role in controlling information processing during both normal and pathological states. Dopamine denervation is the pathological hallmark of Parkinson's disease, while a hyper-dopaminergic state has been associated with hyper-kinetic and hyper-behavioral disorders ranging from dyskinesia to schizophrenia. Within the striatum, dopamine modulates the activity of individual neurons as well as the dynamics within and between the neuronal subpopulations (Murer et al., 2002; Humphries et al., 2009). Phasic and tonic alterations of dopamine levels within the striatum lead to a variety of changes in neuronal activity. Reward related, phasic, release of dopamine from the midbrain leads to inhibition of the cholinergic interneurons (TANs) in both non-human primates and rats, and similar effects has been reported for aversive stimuli (Aosaki et al., 1994b; Raz et al., 1996; Ravel et al., 2003). Chronic depletion of dopamine, such as in the 6-OHDA rat model or MPTP treated primate model of PD leads to an increase in the firing rate of the TANs and the formation of oscillatory firing patterns (Raz et al., 1996; Kish et al., 1999). The effect of dopamine on MSNs has classically been divided into positive modulation via D1-receptors and negative modulation via the D2-receptors (Albin et al., 1989). This basic modulation of the direct (D1 receptor expressing) and indirect (D2 receptor expressing) MSNs is further dependent upon additional factors such as cortical input, as well as the behavioral state of the subject (Moyer et al., 2007; Gerfen and Surmeier, 2011). The FSIs neuronal firing rate has been shown to be dependent on dopamine in that it increases after agonist application and decreases after antagonist application (Wiltschko et al., 2010).

These pronounced motor and behavioral effects of dopamine are utilized in providing pharmacological treatment of multiple motor and psychiatric disorders. Antipsychotic drugs, which are widely used in psychiatric conditions such as psychosis and mania or hyperkinetic motor disorders such as tics and chorea, are classically divided into typical and atypical antipsychotics. Typical antipsychotics (e.g. haloperidol) have a high affinity for D2 receptors (Gardner et al., 2005). The D2 receptor is expressed in the indirect basal ganglia pathway and plays an important role in behavioral, cognitive as well as affective and motor pathologies (Cohen and Frank, 2009). The high affinity of typical antipsychotics for the D2 receptor is known to cause extrapyramidal side effects such as akinesia, dystonia, parkinsonism, and tardive dyskinesia, as well as cognitive and behavioral alterations (Strange, 2008).

The current study assessed the effects of haloperidol on multiple striatal neuron subtypes (MSNs, TANs, and FSIs) in freely moving rats. We used chronic multi-electrode recordings to characterize the changes at the single neuron level and discuss network dynamics within the striatum and their potential role in the cortico-basal ganglia pathway.

## Methods

### Animals

10 adult rats (five Sprague Dawley and five Long-Evans (Harlan) weighing 250–450 g) were used in this study. The animals were kept under controlled temperature, humidity, and lighting (12 h light/dark cycle) conditions and had free access to food and water. Experiments were performed during the light phase of the cycle. All procedures were approved and supervised by the Institutional Animal Care and Use Committee (IACUC) and were in accordance with the National Institute of Health Guide for the Care and Use of Laboratory Animals and the Bar-Ilan University Guidelines for the Use and Care of Laboratory Animals in Research. This study was approved by the National Committee for Experiments in Laboratory Animals at the Ministry of Health (permit number 6-01-11).

### Surgical procedures

After arrival, the animals were housed in their home cage for at least 1 week before the operation. Animals were sedated with 5% isoflurane and then anesthetized with an intra-peritoneal injection of a mixture of ketamine HCl (100 mg/kg) and xylazine HCl (10 mg/kg) and an intra-muscular injection of atropine (0.1 mg). Anesthesia during the operation was maintained using isoflourane and supplementary injections of ketamine. The animals' scalp was shaved and cleaned, and anesthetic cream (2.5% lidocaine and 2.5% prilocaine) was applied into their ears to prevent discomfort during head fixation. The rats were placed in a stereotactic frame (Stoelting Co., Wood Dale, IL, USA) and their head was fixed. The scalp was sterilized and lidocaine (10 mg/ml) was locally injected into the skin around the incision location. A central incision was made and the skull was exposed. The head was then straightened by equalizing bregma-lambda height. Coordinates of the craniotomies were marked and holes were drilled. The dura was removed and 16 electrode arrays were slowly lowered unilaterally into the striatum and were grounded and fixed to the skull by screws and dental cement.

**Figure 1:**
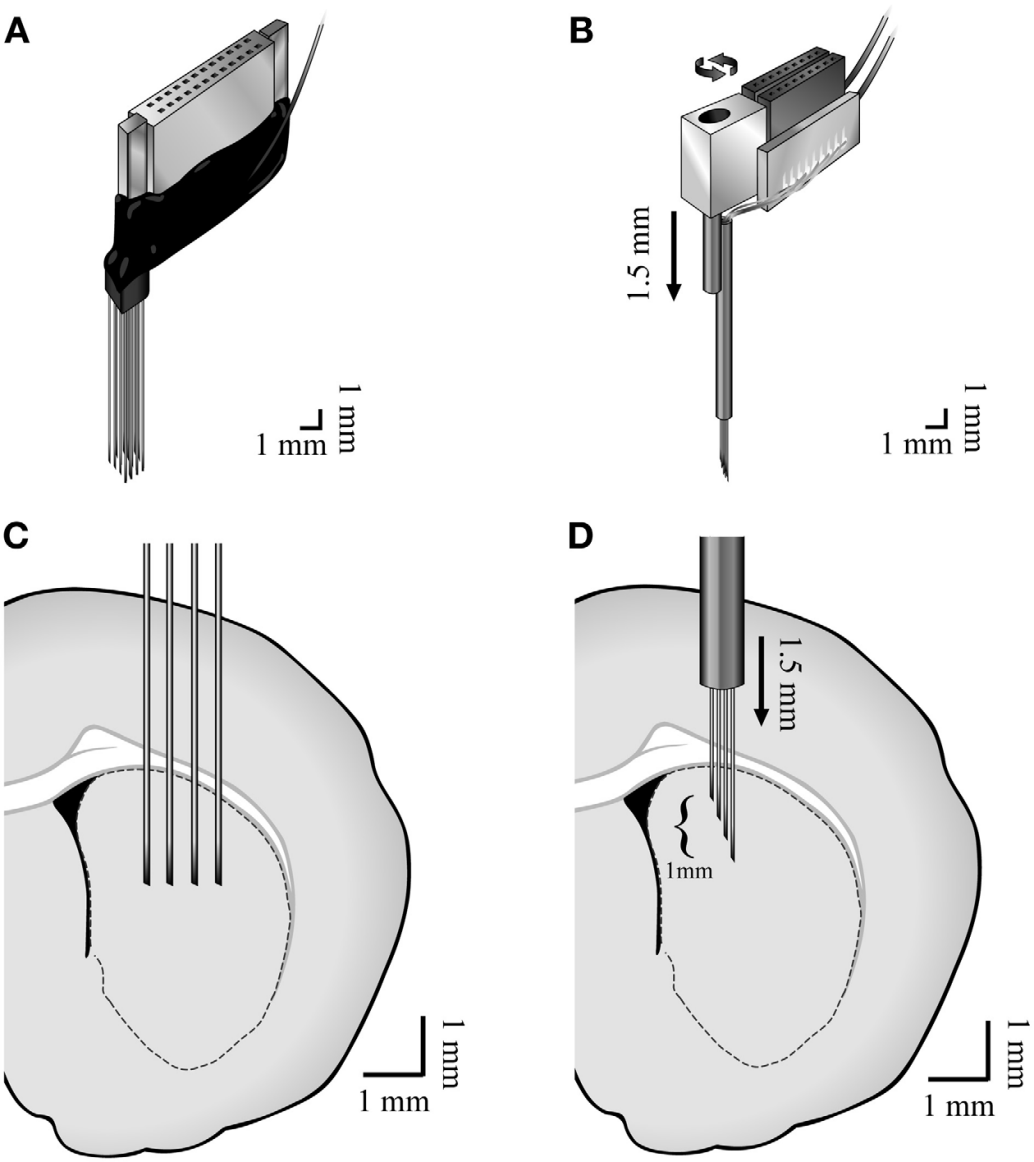
**Methods.** Illustration of the **(A)** non-movable 4 × 4 45 μm diameter isonel-coated tungsten microwire linear arrays and the **(B)** movable bundles of 16 25 μm diameter formvar coated nichrome microwires and **(C,D)** their locations in the dorsal striatum.

Two types of custom made multi-wire arrays were used. Four of the animals were implanted with non-movable 4 × 4 45 μm diameter isonel-coated tungsten microwire linear arrays (Yarom and Cohen, 2011) [AP +1.0, −0.5 ML 2.0, 4.0 DV 5.0 (Paxinos and Watson, 2007)] (Figures 1A,C). These arrays displayed higher probabilities of recording TANs. Six of the animals were implanted with movable bundles of 16 25 μm diameter formvar coated nichrome microwires (Grossman et al., 2008) [AP +1.0, −0.5 ML 2.0, 3.5 DV 4.0 (Paxinos and Watson, 2007)] (Figures 1B,D). These arrays displayed higher probabilities of recording MSNs and FSIs.

### Experimental sessions

Experimental sessions began at least 7 days post-operation to allow full recovery of the animals. The animals were placed in the recording chamber, where they could move freely, and were connected to the recording system. The signal was continuously recorded throughout the session. The analog signal was amplified (^*^200), band-pass filtered (0.5–10000 Hz, 4 pole Butterworth filter) and digitized at a sampling rate of 44 KHz (Alpha-Lab SNR, Alpha-Omega Engineering, Nazareth, Israel). The digitized continuous raw signal was acquired to allow offline sorting and analysis. Sessions initiated with recordings in the naïve state, which was followed by recording of neuronal activity during and after a single subcutaneous haloperidol (0.05 mg) injection. Few experimental sessions were performed in some of the animals (2.5 ± 1.3 mean ± *SD*, sessions/animals), which were separated by prolonged periods (11.3 ± 7 mean ± *SD*, days between sessions) to avoid accumulation of Haloperidol effect. In order to minimize behavioral effects on the results, the electrophysiological recordings took place during resting state of the animals both before and after the injection. Notably, rest state following haloperidol injection included akinesia and rigidity which were not part of the pre-injection state.

### Data preprocessing and analysis

Spike sorting of multiple single units from the continuous raw signal was performed offline (Offline Sorter v2.8.8, Plexon, Dallas, TX). Offline sorting using a continuous signal instead of the more common segmented data makes it possible to obtain long overlapping windows (1.5–2 ms in our data) surrounding each spike, thereby increasing the fidelity and quality of the sorting. Only neurons that formed clear distinguishable clusters were included in the study. Neurons that did not display a stable wave-form throughout the recording session were eliminated from the database. All subsequent analyses were performed using custom-written MATLAB code (Mathworks, Natick, MA).

### Histology

After completion of the experimental sessions, the rats were deeply anesthetized with a mixture of ketamine (100 mg/kg), xylazine (10 mg/kg) and additional morphinhydroclorid (0.15 mg/kg). A current (–100 μA, 10 s) was passed through the tips of the electrodes to create small marker microlesions. Rats were perfused transcardially with 0.9% saline followed by 10% paraformaldehyde. The brains were removed and cryoprotected for a minimum of 48 h in a fixation solution of 30% sucrose in 10% paraformaldehyde. Brains were frozen and cut into 50 μm thick sections on a cryostat. Sections holding the striatum were counterstained with cresyl violet for identification of the structures and electrode placement verification.

## Results

### Experimental sessions

We continuously recorded extracellular activity in the dorsal striatum of freely moving rats in naïve and post-systemic haloperidol injection states using two types of 16 microwire arrays (25 and 45 μm wire diameter). The recordings were made in 10 rats over 22 separate recording sessions. Electrophysiological recordings in the naïve state began after an initial acclimatization period in which the animals were allowed to move freely and explore the recording cage. Recording in the naïve state lasted 30 min, continued throughout the injection time and for a post-injection period of 30 min. Injection of haloperidol induced observable akinesia and rigidity within a few minutes during all of the sessions. These behavioral effects could be modulated for short periods of time; for example, after moving the animals from the recording cage to their home cage, a short period of exploration could occur. An additional common phenomenon occurring after haloperidol injection was increased stereotypical behavior in the form of teeth chattering.

### Neuronal classification

**Figure 2:**
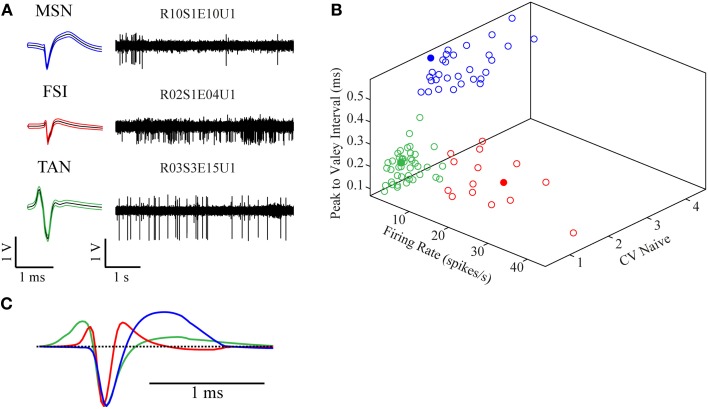
**Neuronal classification into striatal subtypes. (A)** Examples of raw data (right) and spike waveform (left) of MSN, FSI, and TAN (black lines—mean, colored lines—mean ± *SD*). **(B)** Division of the classified neurons into three dimensional clusters by peak to valley width, firing rate and CV in the naïve state. Filled circles are the units presented in **(A)**. **(C)** Mean population waveform of MSN (blue), FSI (red), and TAN (green).

The recorded signal was offline sorted and the dataset of 87 single units was classified into three categories—MSNs (30/87, 34.5%), TANs (42/87, 48.3%), and FSIs (15/87, 17.2%) (Figure 2A). All the neurons presented well separated clusters which were stable throughout the whole recording session. The classification was first done subjectively using multiple parameters such as the mean waveform shape, inter-spike interval (ISI) histogram, firing rate and pattern, and was subsequently verified via clustering using three parameters: (1) mean firing rate during the naïve period, (2) peak to valley width of the mean spike waveform and (3) the ISI coefficient of variation (CV) where the different subpopulations formed three clearly separable clusters (Figure 2B). The mean firing rate was calculated using 600 s long segments taken from the naïve state recordings (ending at least 100 s prior to the haloperidol injection). MSNs typically displayed a low baseline firing rate of 1.9 ± 1.9 Hz (mean ± *SD*), TANs displayed a medium rate 4.2 ± 2.0 Hz (mean ± *SD*) and FSIs displayed the highest baseline rate 17.5 ± 10.3 Hz (mean ± *SD*). The CV was calculated using the same time windows by dividing the standard deviation of the 1st order ISIs by their mean. This measure varied across the striatal subpopulations: MSNs typically displayed a bursty firing pattern over low baseline activity and high CV value of 2.5 ± 0.7 (mean ± *SD*). FSIs displayed a bursty firing pattern over high baseline activity and a CV of 1.7 ± 0.6 (mean ± *SD*). TANs displayed a Poisson-like firing pattern and a CV value of 0.9 ± 0.3 (mean ± *SD*). Waveform peak to valley width was calculated using the waveforms of the whole recording periods. MSNs waveforms were typically wide and displayed a long positive second phase. FSIs displayed a narrow waveform, and TANs displayed wide waveform containing initial positive phase (Figure 2C).

### Haloperidol effects

Different statistics for comparison of the naïve, pre-haloperidol injection state and the post-haloperidol injection state were calculated using stable periods of continuous 600 s recordings from the naive state, ending at least 100 s prior to the haloperidol injection, and from the post-haloperidol injection state, starting at least 600 s after the haloperidol injection. This procedure produced stable periods and served to avoid transition periods.

**Figure 3:**
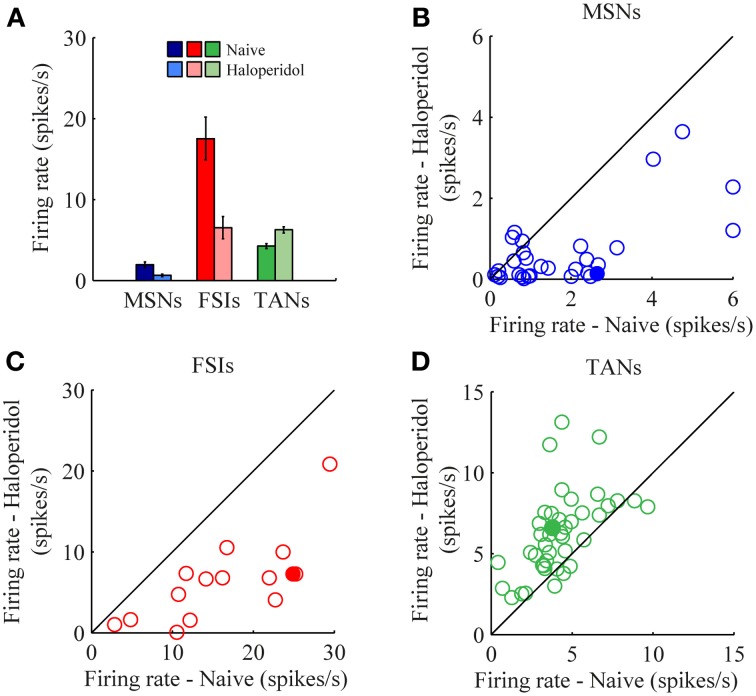
**Haloperidol-induced firing rate changes. (A)** Population mean firing rates, before and after haloperidol injection. Error bars—s.e.m. Single neuron firing rates in the naïve and post-haloperidol injection states of **(B)** MSNs, **(C)** FSIs, and **(D)** TANs. Filled circles—the neurons shown in (Figure 4A).

**Figure 4:**
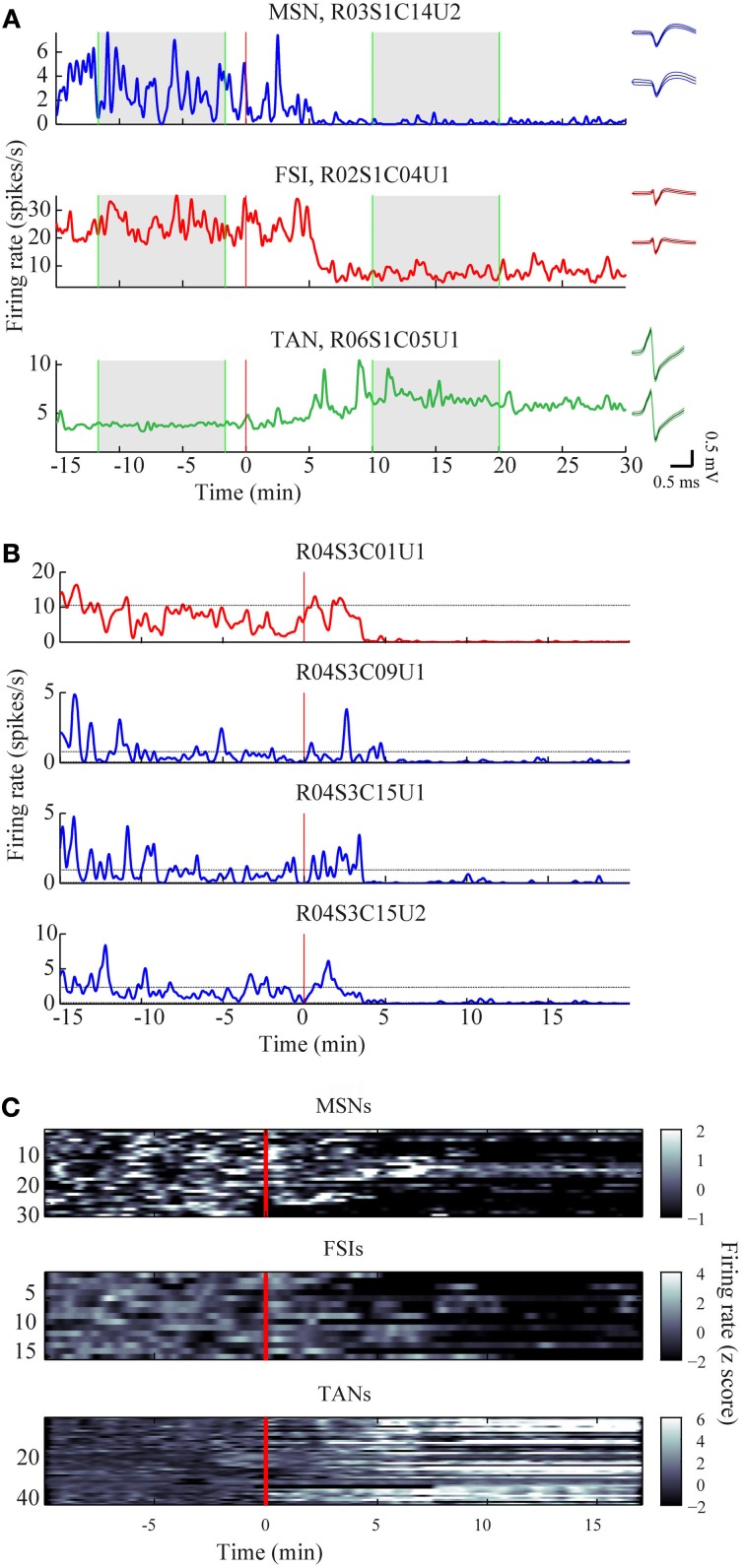
**Time course of firing rate modulations. (A)** Examples of firing rate modulations of single MSN, FSI, and TAN. Red vertical lines—haloperidol injection times. Right panel—waveforms of the units before (top) and after (bottom) haloperidol injection (black lines—mean, colored lines mean ± *SD*). Green vertical lines—time frames used for stable states calculations. **(B)** Firing rate modulation of the whole neuronal population represented as z scores. **(C)** Example of haloperidol—induced firing rates modulations of one FSI (top) and three MSNs (bottom) recorded simultaneously.

The mean firing rates of the neurons during the stable windows of the naïve and post-haloperidol states were compared. Both MSNs and FSIs significantly lowered their firing rates after haloperidol injection (paired *t*-test, *p* < 0.01) from the mean population baseline rates of 1.9 ± 0.4 Hz (mean ± s.e.m.) and 17.5 ± 2.6 Hz (mean± s.e.m.) to firing rates of 0.6 ± 0.2 Hz (mean ± s.e.m.) and 6.5 ± 1.4 Hz (mean ± s.e.m.), respectively (Figure 3A). To assess the behavior of individual neurons within each subtype comparisons were made of the distribution of firing rates of each neuron in 60 short (10 s) windows making up the stable periods. In a single unit analysis, 80% (24/30) of the MSNs (Figure 3B) and 100% (15/15) of the FSIs (Figure 3C) significantly lowered their firing rates after the haloperidol injection (*t*-test, *p* < 0.01). The TANs' firing rates were elevated after the haloperidol injection (paired *t*-test, *p* < 0.01) from a mean population baseline rate of 4.2 ± 0.3 Hz (mean ± s.e.m.) to a firing rate of 6.3 ± 0.4 Hz (mean ± s.e.m.) (Figure 3A). In a single unit analysis, 88% (37/42) of the TANs elevated their firing rate post-haloperidol injection in a significant manner (*t*-test, *p* < 0.01) (Figure 3D). The transition dynamics of firing rates following haloperidol were complex and dependent on the specific animal and session, however, the effects were clear across the population (Figures 4A,B). Despite the different dynamics across the sessions, simultaneously recorded neurons demonstrated the same dynamics in their transition phases and timing following the injection (Figure 4C).

**Figure 5:**
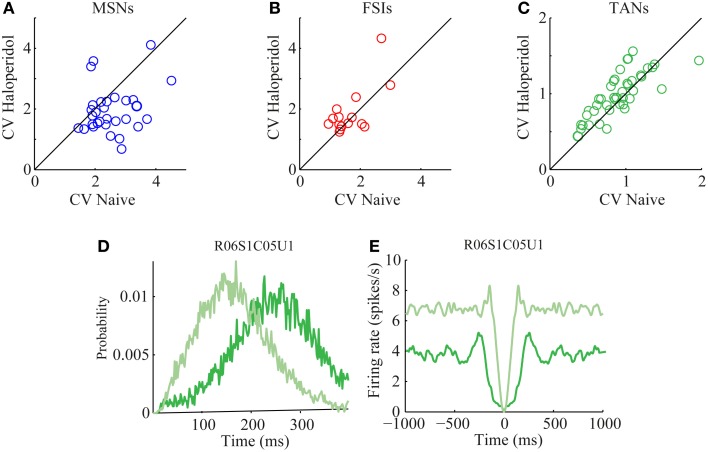
**Haloperidol—induced firing patterns modulations.** Single neuron CVs in the naïve and post-haloperidol injection states of **(A)** MSNs, **(B)** FSIs, and **(C)** TANs. Example of **(D)** ISI histogram and **(E)** autocorrelation function of a single TAN (dark green—naïve state, light green—post-haloperidol state).

The CV of the naïve and post-haloperidol states were calculated for single units during the same 600 s stable time frames by dividing the standard deviation of the ISIs by their mean. The MSNs significantly (paired *t*-test, *p* < 0.01) lowered their CVs following the haloperidol injection from a value of 2.53 ± 0.13 (mean ± s.e.m.) to a value of 1.95 ± 0.14 (mean ± s.e.m.) (Figure 5A). CV values of FSIs population did not change significantly (naïve 1.7 ± 0.2, post-haloperidol 1.9 ± 0.2, paired *t*-test *p* > 0.01) (Figure 5B). TANs significantly (paired *t*-test, *p* < 0.01) elevated their CV values from a baseline value of 0.90 ± 0.05 (mean ± s.e.m.) to a value of 0.99 ± 0.05 (mean ± s.e.m.) post-haloperidol injection (Figure 5C). The changes in the TANs' CV values following the haloperidol injection might be explained by the shortening of their refractory period, which was clearly seen in their ISI histograms (Figure 5D) and autocorrelation functions (Figure 5E).

**Figure 6:**
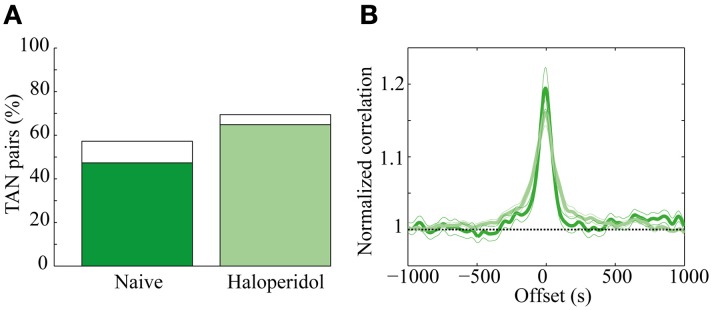
**Haloperidol modulation of TAN correlations. (A)** Cross-correlation between TAN pairs, divided into significant (*p* < 0.01) positively correlated (green) and negatively correlated (white). **(B)** Mean correlation of the TAN pairs in the naïve (dark green) and post-haloperidol (light green). The narrow lines indicate one s.e.m. significance lines and the horizontal black dashed line indicates the normalized unity line. The scale is normalized to the mean rate of each pair.

A total of 131 of TAN pairs were recorded simultaneously in the naïve and in the subsequent post-haloperidol injection state. In the naïve state many pairs exhibited significant (*p* < 0.01) positive correlations (62/131, 47.3%) and a smaller minority had significantly negative correlations (13/131, 9.9%). Following the haloperidol injection the asymmetry increased, in that the majority of the neurons displayed a significant positive correlation (85/131, 64.9%) and a smaller minority showed significant negatively correlations (6/131, 4.6%) (Figure 6A). The haloperidol injection changed the fraction of significantly correlated neurons in a significant manner (χ^2^ test, *p* < 0.01). However, the mean pair-wise correlation, which was highly positive in both the naïve and post-haloperidol states, did not differ between the two states (Figure 6B). This correlation was calculated on the rate normalized correlation functions. Using the raw cross correlation functions yielded larger positive correlations, where the skew was due to the increased firing rate during the post-haloperidol state.

**Figure 7:**
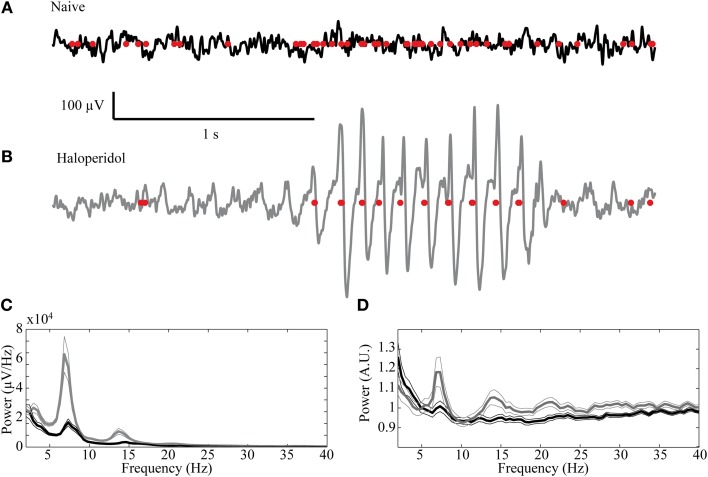
**High voltage spindles (HVSs) in the striatum.** Example of a recording trace of the LFP (black line—naïve, gray—post-haloperidol injection) overlaid with the concurrent spikes (red dots) during the **(A)** naïve state and **(B)** post-haloperidol injection reflecting the HVSs. **(C,D)** Mean power of the **(C)** LFP recording (*n* = 72) and **(D)** spike trains (*n* = 87) during the naïve state (black) and post-haloperidol state (gray). The narrow lines indicate one s.e.m.

Oscillatory activity in the 7–9 Hz band could be observed in some of the LFP recordings in the naïve state. These oscillations, which are typical of high voltage spindles (HVS), occurred in 19/72 (26.4%) of the recordings and were typically short and intermittent. Following haloperidol injection the number of oscillatory LFPs increased significantly to 33/72 (45.8%) (χ^2^ test, *p* < 0.01) and their duration and abundance increased (Figures 7A,B). The mean power of the LFP increased preferentially in the HVS frequency band and its harmonics (Figure 7C). This was accompanied by a smaller but significant peak in the HVS frequency and its first harmonic in the spiking activity of single neurons (Figure 7D). The number of neurons with a significant peak in the power spectrum increased from 7/87 (8.1%) in the naïve state to 21/87 (24.1%) during the post-haloperidol state (χ^2^ test, *p* < 0.01).

**Figure 8:**
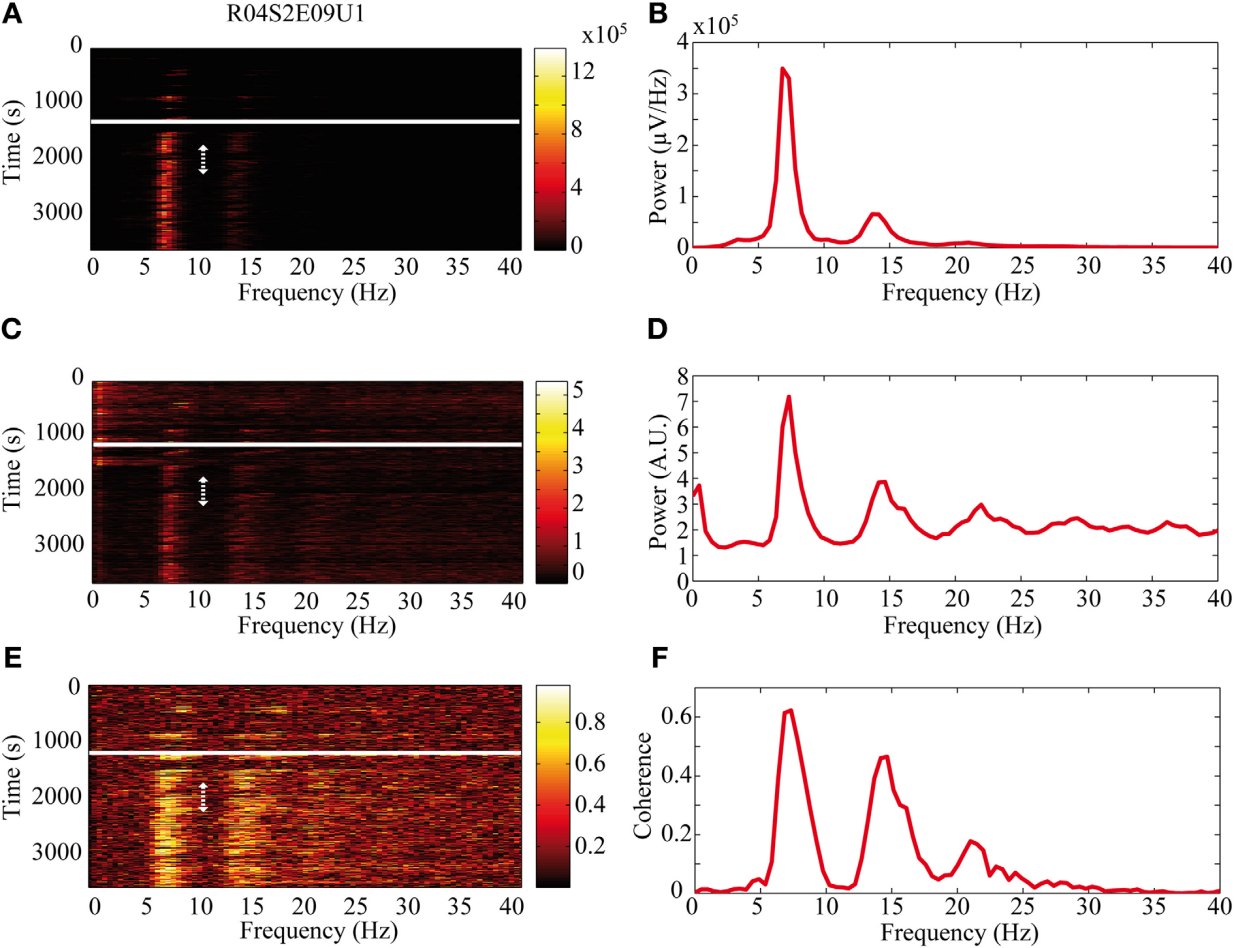
**Oscillatory neuronal activity during HVSs.** The oscillatory activity is shown for a single recording of an FSI which spikes in an oscillatory manner during HVSs: **(A,B)** the LFP, **(C,D)** the spiking activity of a neuron sorted from the same electrode and **(E,F)** the coherence between the LFP and the spiking activity. The **(A,C)** spectrogram and **(E)** coherogram illustrate the changes in the oscillatory activity of the signals over 10 s windows throughout the session. The solid white line indicates the haloperidol injection time and the dotted white arrow indicates the stable post-haloperidol segment. The mean **(B,D)** power spectrum and **(F)** coherence are averaged over the stable post-haloperidol segment.

**Figure 9:**
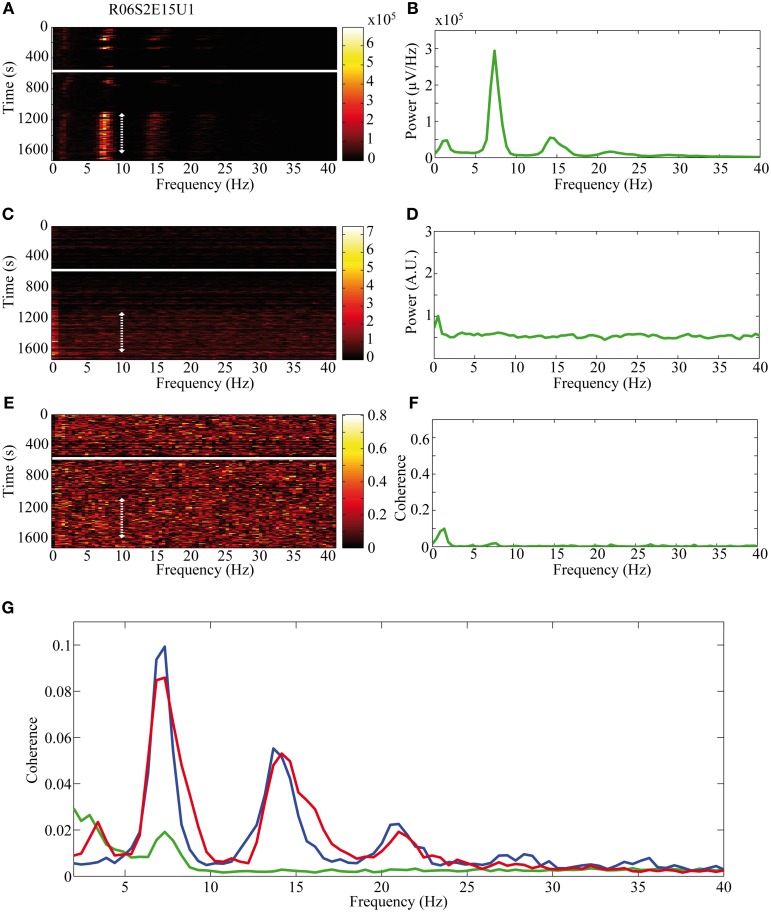
**Non-oscillatory neuronal activity during HVSs.** The oscillatory activity is shown for a single recording of a TAN which does not spike in an oscillatory manner during HVSs: **(A,B)** the LFP, **(C,D)** the spiking activity of a neuron sorted from the same electrode and **(E,F)** the coherence between the LFP and the spiking activity. The **(A,C)** spectrogram and **(E)** coherogram illustrate the changes in the oscillatory activity of the signals over 10 s windows throughout the session. The solid white line indicates the haloperidol injection time and the dotted white arrow indicates the stable post-haloperidol segment. The mean (**B,D)** power spectrum and **(F)** coherence are averaged over the stable post-haloperidol segment. **(G)** Mean coherence between the LFP signal and the spiking activity of MSNs (blue), FSIs (red), and TANs (green) during the post-haloperidol segment.

The activity of single neurons during HVS varied greatly depending on the neuronal sub-type. Both FSIs and MSNs fired in an oscillatory manner in a fixed phase with the LFP. Oscillations of the LFP in the HSV frequency appeared prior to the injection but were increased notably following the haloperidol injection (Figures 8A,B). This was accompanied by equivalent oscillations in the neuronal spiking activity of MSNs and FSIs (FSI example—Figures 8C,D). The LFP and spike train signals were highly coherent following the haloperidol injection in the HVS base frequency and its harmonics (Figures 8E,F). In sharp contrast, TANs did not display spike train oscillatory activity during oscillations in the LFP (Figures 9A–F) and thus were not coherent with the LFP signal. This property was evident when observing the overall neuronal population, which demonstrated a roughly equal coherence of MSNs and FSIs with their background LFP with only a small coherence for the TAN population (Figure 9G).

## Discussion

In this study, we examined the effect of systemic haloperidol administration on the activity of neurons from different subpopulations within the striatum in freely moving rats. Our results demonstrate that neuronal sub-types within the striatum responded in a distinctly different fashion to the haloperidol application. MSNs and FSIs dramatically lowered their firing rate following haloperidol injection, whereas changes in the firing pattern were seen in the MSNs, which became more regular as a result of decreased bursting. In addition, the low frequency oscillations (7–9 Hz) that are typical of HVSs were evident following the haloperidol injection where the LFP signal became highly oscillatory and the firing pattern of both FSIs and MSNs became phase locked to these oscillations. The firing rate of TANs, on the other hand, showed a drug-induced increased firing rate, accompanied by a more irregular firing pattern resulting from a shortening of the relative refractory period. The TANs displayed a non-oscillatory firing pattern during periods of HVS and maintained the typical widely correlated activity within the sub-population.

The effects of dopamine modulation on both behavior and its underlying neuronal mechanisms have been studied extensively in the cortico-basal ganglia loop. Multiple techniques for dopamine modulation have been used in the study of movement and psychiatric disorders such as Parkinson's disease, Huntington's disease and Tourette syndrome, as well as in the study of normal processes such as plasticity, learning and reward. These techniques differ in such key aspects as (1) spread, (2) specificity, and (3) duration of the effect, which makes comparison across results complex. Dopamine denervation techniques range from widespread, non-specific chronic depletion of dopamine neurons, such as those observed following 6-OHDA injections or with genetic knockout models, to acute, focal, and specific antagonisms caused by localized dopamine antagonist microinjections or focal optogenetic modulations. In the current study we used systemic acute injections of the D2 antagonist haloperidol. In this case, spatially, the effect was widespread in the brain and did not only influence the striatal neurons. However, temporally, it had a limited duration, with minor impact on long term changes in the network function and structure. By contrast, chronic treatment with haloperidol has been shown to lead to significant plastic changes (Benes et al., 1985) and behavioral symptoms resembling tardive dyskinesia (Kinon et al., 1984). In addition, the dosage of haloperidol used in this study (0.05 mg) was lower by an order of magnitude than some recent studies (Burkhardt et al., 2007; Yang et al., 2013) that have examined its effect on the basal ganglia. The low dosage elicited the same behavioral symptoms to a large extent, while avoiding permanent damage such as potential apoptosis in the striatum and SNr (Mitchell et al., 2002) and resembling the dosage used in human patients.

Despite the differences between techniques, dopamine denervation mostly yields similar behavioral and neuronal results. Behaviorally, decreased dopamine levels have been shown to induce akinesia and parkinsonian-like symptoms such as rigidity. These outcomes are common to studies using localized 6-OHDA injection (Deumens et al., 2002), systemic injections of dopamine antagonists (Burkhardt et al., 2007) and tetrodotoxin (TTX) injections to the medial forebrain bundle (MFB) (Prosperetti et al., 2013). Neurophysiologically, striatal activity has been widely studied. However, the classification of striatal neurons into different populations has only recently become standardized, leaving multiple early studies hard to interpret as their data contain a myriad of cell types.

TANs in the dorsal striatum express both D2 and D5 receptors and receive dopaminergic input primarily from the SNc (Levey et al., 1993). *In vivo* intracellular recordings have shown that dopamine increase, via stimulation of the SNc, induces inhibition of the firing rate of TANs (Reynolds et al., 2004). Tonic dopamine depletion in 6-OHDA rats, on the other hand, increases their baseline discharge rate (Kish et al., 1999; Sanchez et al., 2011). Similarly, an acute block of the MFB by TTX also increases the firing rate (Prosperetti et al., 2013). Interestingly, while apomorphine likewise suppresses the firing rates of TANs (Fujita et al., 2013), D-Amphetamine, which increases the release of dopamine and inhibits its re-uptake, leads to an increased firing rate of TANs (Kish et al., 1999). In the non-human primate, no effect on spontaneous discharge rate of TANs by localized microinjections of either a D2 antagonist (sulpiride) or a D1 antagonist (SCH23390) (Watanabe and Kimura, 1998), or by unilateral dopamine depletion by MPTP infusion to the caudate-putamen complex (Aosaki et al., 1994a), was observed in the striatum. Generally, the increased rate of TANs following haloperidol injection in our study is in line with most of the literature of dopamine modulation.

There are few studies of FSI modulation by dopamine given the nascent state of the field. However, the decreased firing rate of the FSIs following haloperidol application is consistent with *in vivo* studies reporting a decreased firing rate post eticlopride, a D2 receptor antagonist (Wiltschko et al., 2010).The effect of D2 on FSIs has been hypothesized to result from modulation of their GABAergic input (Bracci et al., 2002).

Unlike the general consistency between reports on TAN and FSI activity following dopamine modulations, previous studies of MSN activity paint a diverse and complex picture. The range of expression of dopamine receptors on MSNs is diverse: D1-like receptors have classically been assigned to the direct striato-nigral pathway and the D2-like receptors to the indirect striato-pallidal pathway and their activation increases and decreases the firing rate of MSNs, respectively (Albin et al., 1989). This might partially explain the heterogeneous results on the activity of MSNs following dopamine modulation in studies which do not break down MSNs into different subpopulations or use specific receptor modulation (Prosperetti et al., 2013). Chronically denervated animals tend to show an overall increase in firing rate (Kish et al., 1999; Chen et al., 2001), whereas acute denervation leads to increases and decreases in firing rate (Wiltschko et al., 2010; Prosperetti et al., 2013). Our data show contradictory results since most of our MSNs significantly lowered their firing rate following haloperidol administration. This is a surprising result in that the D2 receptor inactivation is expected to increase the MSN firing rate (Albin et al., 1989; DeLong, 1990), which may result from indirect network interactions.

The relationship between FSIs and MSNs firing following haloperidol injection did not follow their presumed interaction. A decrease in the firing rate of FSIs is expected to elevate the MSN firing rate, due to the strong inhibition that FSIs exert on MSNs (Koos and Tepper, 1999) whereas in our study both FSIs and MSNs lowered their firing rate. This discrepancy has been reported elsewhere, but the mechanism is still under debate (Lansink et al., 2010; Wiltschko et al., 2010). The most common suppositions are that in the dopamine depleted state, the FSIs reduce their sensitivity to cortical stimulation or the cortico-striatal input might have a larger influence post dopamine depletion (Kimura, 1990; Sharott et al., 2009; Berke, 2011).

HVSs appear in awake immobile rats and are characterized by a typical frequency of 5–13 Hz with a typical spike and wave shape (Klingberg and Pickenhain, 1968). HVSs have been studied extensively in multiple brain areas and have been observed in different parts of the cortico-basal ganglia loop. The origin of HVSs is still under debate and thalamic as well as cortical influences are thought to play an important role (Berke et al., 2004). Neurons in the striatum (presumably MSNs) were shown to fire in a phase locked with HVS, mostly within the spike component (Buzsaki et al., 1990). HVSs were shown to affect neuronal activity in the basal ganglia input (striatum—MSNs) and output (SNr) which are phase-locked to both the cortical and basal ganglia local field potentials (Dejean et al., 2007). Dopamine modulates HVSs, and recent studies have demonstrated the significant increase in these oscillations following dopamine depletion and specifically the LFP oscillations in the cortex and GP following systematic application of D2 antagonists (Yang et al., 2013). Under the dopamine depletion state in the 6-OHDA rat, the duration and frequency of occurrence of HVSs increased significantly (Dejean et al., 2008). This increase was also apparent following the injection of dopamine antagonists directly to the striatum (Deransart et al., 2000). Our results extend previously reported work by revealing that the effect of HVSs is not uniform across the different neuronal subpopulations. Whereas haloperidol increases the abundance of HVSs, which is consistent with the literature regarding dopaminergic reduction in the striatum and triggers FSIs and MSNs to show phase-locked activity to the LFP, TANs do not reveal this phenomenon and remain largely non-oscillatory. This contrasts with previous studies in the parkinsonian primate, which have found oscillatory activity that was coherent with basal ganglia oscillations (Raz et al., 1996, 2001).This might be explained by an intrinsic ability to generate spikes of TANs and/or stronger cortical or thalamocortical influences on MSNs and FSIs.

Haloperidol, given at amounts comparable to the dosage administered to human patients suffering from different movement and psychiatric disorders, produces profound behavioral and neuronal effects. Despite the clear behavioral effect, the underlying neuronal mechanism appears complex and diverse even within only the striatum itself. Different sub-populations of neurons are affected differentially and the effects include changes in neuronal rate, pattern, and interactions. This emphasizes the need to generate fine grain clustering of neurons to elucidate the mechanisms of action of pharmaceuticals on the brain as a whole and specifically on the striatum.

### Conflict of interest statement

The authors declare that the research was conducted in the absence of any commercial or financial relationships that could be construed as a potential conflict of interest.

## Abbreviations

BG: basal ganglia
FSI: fast spiking interneuron
GP: globus pallidus
HVS: high voltage spindle
MSN: medium spiny neuron
SNc: substantia nigra pars compacta
TAN: tonically active neuron.
